# Survey of ectoparasites affecting dog and cat populations living in sympatry in Gamo Zone, Southern Ethiopia

**DOI:** 10.1002/vms3.1413

**Published:** 2024-03-20

**Authors:** Hana Taddesse, Marika Grillini, Dinka Ayana, Antonio Frangipane di Regalbono, Rudi Cassini, Bersissa Kumsa

**Affiliations:** ^1^ Southern Agricultural Research Institute Arba Minch Agricultural Research Center Arba Minch Ethiopia; ^2^ Department of Veterinary Parasitology and Pathology Addis Ababa University Bishoftu Ethiopia; ^3^ Department of Animal Medicine Production and Health University of Padova Padova Italy

**Keywords:** cat flea, dog, Ethiopia, Gamo Zone, lice, tick

## Abstract

**Background:**

Ectoparasites of dogs and cats are implicated to be responsible for life‐threatening anaemia, allergic dermatitis and pruritic and non‐pruritic skin disorders. In Ethiopia, few studies have been conducted on the arthropods of dogs and cats.

**Objectives:**

In order to shed light on some of these aspects, a survey was conducted to investigate the presence of ectoparasites in dogs and cats living in sympatry in the urban and rural areas of Gamo Zone, Ethiopia.

**Methods:**

A total of 297 dogs and 110 cats were examined for ectoparasites, and questionnaires were employed to obtain information concerning owner's knowledge about arthropods and vector‐borne diseases (VBDs).

**Results:**

The overall prevalence of ticks, fleas and lice in dogs was 36.7%, 69.7% and 4.7%, respectively. Similarly, on cats, an overall prevalence of 2.7% ticks and 21.8% fleas was recorded. On dogs, fleas (*Ctenocephalides felis* 69.4%, *Echidnophaga gallinacea* 1.3%, *Ctenocephalides canis* 1.0% and *Pulex irritans* 0.3%), ticks (*Amblyomma variegatum* 22.9%, *Rhipicephalus sanguineus* 14.1%, *Haemaphysalis leachi* 8.8%, *Rhipicephalus praetextatus* 4.0% and *Rhipicephalus pulchellus* 3.4%) and lice (*Heterodoxus spiniger* 4.0% and *Trichodectes canis* 0.7%) were identified. Likewise, on cats, fleas (*C. felis* [15.5%] and *E. gallinacea* [7.3%]) and ticks (*H. leachi* [2.7%]) were identified. The abundance of *C. felis* was significantly higher (*p* < 0.001) on dogs, whereas in cats, the abundance *of E. gallinacea* was significantly higher (*p* = 0.002) than the other ectoparasites. On dogs, a significantly higher prevalence of *Rh. sanguineus* was recorded in urban areas (<0.001) and on dogs which live in indoor environments (*p* = 0.003) than on dogs which live in other environments. On the other hand, the prevalence of *A. variegatum* in rural areas and midland agroecology was significantly higher (*p* < 0.001). The prevalence of *H. leachi* was significantly higher in midland (*p* < 0.001) and on adult dogs (*p* = 0.001). Overall, fleas were more prevalent in rural (*p* = 0.029) than in urban areas, and female dogs were with higher infestation than the male (*p* = 0.047) dogs; *C. felis* was prevalent in female (*p* = 0.038) dogs than males. Overall, 88.3% owners in the study area had no knowledge about ectoparasites and VBDs of dogs and cats. Majority of the owners (64.8%) attest that they had never visited veterinary clinics.

**Conclusions:**

In conclusion, the data presented in the present study provide additional knowledge on the importance of ectoparasites of dogs and cats and are believed to contribute in awareness creation and strengthening of veterinary services of dogs and cats of the study area.

## Graphical abstract

Findings of the present study demonstrated an overall prevalence of 36.7%, 69.7% and 4.7% ticks, fleas and lice on dogs, respectively. Similarly, on cats, an overall prevalence of 2.7% ticks and 21.8% fleas was recorded. The abundance of *C. felis* was significantly higher (*p* < 0.001) on dogs, whereas in cats, the abundance of *E. gallinacea* was significantly higher (*p* = 0.002) than the other ectoparasites.

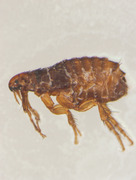

## 1 INTRODUCTION

Ectoparasites, such as ticks, fleas and lice, have been reported on dogs and cats worldwide (Stork, 2018). In dogs and cats, ectoparasites are implicated to be responsible for life‐threatening anaemia, allergic dermatitis and pruritic and non‐pruritic skin disorders (Omonijo & Sowemimo, 2017). In addition, ectoparasites play great role in the transmission of pathogens of great veterinary and public health significance. Some ectoparasites can act as vectors of different pathogens, such as *Babesia*, *Bartonella* and *Rickettsia* species (Alcaíno et al., 2002; Heukelbach et al., 2012; Nuchjangreed & Somprasong, 2007). Vectors and vector‐borne diseases (VBDs) are very common throughout Africa, including Ethiopia. Ectoparasites and their vectored pathogens have been well studied in livestock of Sub‐Saharan Africa but poorly investigated in dogs and cats (Heylen et al., 2021).

Several previous studies have confirmed that ectoparasites, such as ticks, fleas and lice, are common ectoparasites of domestic dogs and cats in tropical countries (Abdulkareem et al., 2018; Siagian & Siregar, 2021; Tadesse et al., 2019). However, to date, the role of dogs and cats in the epidemiology of ectoparasites and VBDs in Sub‐Saharan Africa has not been addressed appropriately. In recent decades, the socio‐economic value of dogs and cats has increased in this area (Heylen et al., 2021; Otranto & Wall, 2008). Besides, human‐mediated environmental alterations (i.e. expansion of farmland, urbanization, deforestation and establishments of settlements in natural ecosystems) give rise to an increased risk of infestation by ectoparasites and, thus, exposure to VBDs and new opportunities for novel transmission cycles (Heylen et al., 2021).

In Ethiopia, few studies have been conducted on ectoparasites of dogs and cats (Kumsa & Mekonnen, 2011; Kumsa et al., 2019; Tesfaye & Chanie, 2011; Tadesse et al., 2019), and none has been undertaken to date in the present study area in Gamo Zone, Southern Ethiopia. This study was, therefore, aimed to provide information on the ectoparasite species and prevalence in dogs and cats in the urban area of Arba Minch town and in selected neighbouring urban and rural districts (typical areas with expanding farming and urbanization). Additional purpose was to investigate the potential risk factors to make hypothesis on the epidemiological role played by these two sympatric host species in the maintenance of ectoparasites and potential role for VBDs.

## 2 METHODOLOGY

### 2.1 Study area

Gamo Zone is located in Southern Nations, Nationalities, and People's Regional State between 5°55′N and 6°20′N latitude and between 37°10′E and 37°40′E longitude. Arba Minch town, the capital of Gamo Zone, is 502 km south of Addis Ababa. In 2014, the total population of the zone was estimated to be 1901,953 (Central Statistical Agency [CSA], 2013). Elevation ranges between 600 and 4207 m above sea level, and it covers an area of 6735 km^2^. The annual average temperature ranges from 15 to 28°C, whereas the mean annual rainfall ranges from 200 to 2000 mm. The rainfall pattern can be characterized as a bimodal minor rainy season (September–November) and the major rainy season (March–May). The main rainy season accounts for 70%–90% of the total annual rainfall. Air temperature largely depends on the altitude; it decreases with increasing altitude. Most of the natural vegetation consists of woodland and Savannas. In the highlands, Afromontane forests are found (Shalishe et al., 2022). Sampling was carried out between November 2020 and January 2021 in four districts of Gamo Zone in Southern Ethiopia: Arba Minch town, Chencha town, Arba Minch Zuria and Gerese. The districts and households were selected purposively, being the ones with higher dog and cat populations; Arba Minch Zuria district elevation ranges from a warm semiarid midland (1200 masl) around eastern part to a cool and humid highland (3000 masl) in north‐western part near Chencha town. Arba Minch town has a warm semiarid climate, which is lowland and lies at an altitude of 1285 masl. Gerese district lies in an altitude of 2400 masl, which has also cool and humid highland characteristics. Chencha town is located at an altitude of 2732 masl. The territory of the Zone is divided into three agroecology zones, named in local language Dega (highland), Woinadega (midland) and Kolla (lowland), on the basis of their climatic/geographic aspects. All agroecological classifications were done based on Ethiopian Ministry of Agriculture guidelines (MoA, 1998).

### 2.2 Study population

A total of 297 dogs and 110 cats were identified for ectoparasite collection. All the study dogs and cats were included, irrespective of their sex, age and life style. In this study, apparently healthy owned dogs and cats were included. Ectoparasite collection and questionary survey

A cross‐sectional study design was used to assess ectoparasites of owned dogs and cats through house‐to‐house survey based on owners’ willingness to participate. A structured questionnaire was conducted within each household to collect information on the general knowledge of dog and cat owners about ectoparasites and VBDs, according to Arsham (2005). Overall, 290 dog/cat owners were included in this study and interviewed, specifically 143 from Arba Minch town, 85 from Arba Minch Zuria district, 49 from Gerese district and 13 from Chencha town.

Ectoparasites were collected from dogs and cats as described below. An anamnestic sheet was compiled for each sampled animal, including information on the species, sex, age (in months) and life style. Age was estimated by dental formulary and owner information. The agroecological zone and the urban or rural characteristics of the area were decided according to the location of the household (district and Peasant Association (PA) administrations).

The skins of all dogs and cats were palpated and visually inspected thoroughly for the presence of ticks, lice and fleas by going through all parts of the body for 10–15 min. All ticks were removed carefully to ensure that the mouth‐parts remained intact by using forceps. For the collection of lice and fleas, each animal was combed using a standard fine metal flea comb (12 teeth per 1 cm) for 10–15 min (Kumsa & Mekonnen, 2011; Marchiondo et al., 2007). After combing, the flea comb was held over a white plastic tray, and fleas and lice were collected with forceps from the tray. All the collected ticks, lice and fleas from each dog and cat were moved into labelled specimen bottles containing 70% ethanol and subsequently transferred to the laboratory.

### 2.3 Ectoparasite identification

Ectoparasite specimens from all dogs and cats were counted, recorded and identified thoroughly in the laboratory of Addis Ababa University, College of Veterinary Medicine and Agriculture and Parasitology Laboratory. Each ectoparasite was identified at genus or species level, and their sex was determined under a microscope according to the descriptions of Hoogstraal (1956), Okello‐onen et al. (1999) and Walker et al. (2013, 2000) for ticks, and Wall and Shearer (1997) and Taylor et al. (2007) for lice and fleas. Adult ticks were identified at species level, whereas larvae and nymphs were identified only at genus level (Hoogstraal, 1956; Walker et al., 2013, 2000).

### 2.4 Data analysis

Descriptive statistics (i.e. prevalence and mean abundance values) was used to summarize and display in tables the level of infestation for each group of ectoparasites (fleas, ticks and lice) for both dog and cat species.

The Pearson Chi‐square (*X* ^2^) test, or Fisher's exact test when appropriate, was applied to assess significant differences between dog and cat infestation rates for the different parasite groups and species. Similarly, within each of the two host species, the same approach was used to assess differences in groups identified by age class, sex, life style, urban/rural areas and agroecology. Concerning dogs, age classes include young (up to 12 months) and adult (older than 1 year), whereas life style was categorized in indoor (if the dog had access to indoor also partially) and outdoor (if dog was always outdoor). Likewise, cats were categorized as previously described in young (up to 6 months) and adult (older than 6 months) (Kumsa & Mekonnen, 2011; Thomas et al., 2016), and the life style differentiated in indoor (if the cat was always indoor) and outdoor (if the cat had access to outdoor also partially). The urban or rural environment and the agroecology zone were determined based on information provided by local administrations and by the Ethiopian Ministry of Agriculture Agroecological Zone classification.

Differences in the infestation burden between dogs and cats were investigated only for relevant groups or species of parasites (i.e. for the ones with higher abundance values), by means of a non‐parametric approach (Mann–Whitney *U*‐test). Data analysis was performed with IBM SPSS Statistics for Windows, version 28 (IBM Corp.). Differences were considered significant at *p *< 0.05.

## 3 RESULTS

Two‐hundred fifty‐six (88.3%) dog/cat owners had no knowledge about arthropods and VBDs of dogs and cats. Only one third of the interviewed persons had ever visited a veterinary clinic, and most of the dog owners visited veterinary clinics for rabies vaccination (*n* = 96, 33.1%) (Table 1).

**TABLE 1:** Results of the questionnaire survey on general characteristics of pet owners (*n* = 290).

Variables	Category	*N* of owners (%)
Age	7–25	46 (15.9)
	26–55	197 (67.9)
	>55	47 (16.2)
Gender	Male	231 (79.7)
	Female	59 (20.3)
Education	Illiterate	82 (28.3)
	Elementary	92 (31.7)
	High school	53 (18.3)
	College	62 (21.4)
	Spiritual	1 (0.3)
Knowledge about VBDs	Yes	34 (11.7)
	No	256 (88.3)
Person infected in the household	No	256 (88.3)
	*Leishmania* (cutaneous)	1 (0.3)
	Relapsing fever	1 (0.3)
	Typhus	31 (10.7)
	Typhus and *Leishmania*	1 (0.3)
Purpose of having dog/cat	Companion and guard	2 (0.7)
	Companion and rat protection	100 (34.5)
	Guard	188 (64.8)
Pet origin	Breeding in the house	69 (23.8)
	Neighbour	176 (60.7)
	Other places	45 (15.5)
Reason for Vet. clinic visit	No visit	188 (64.8)
	Ectoparasite infestation	2 (0.7)
	Infection and stomach upset	2 (0.7)
	Loss of appetite and weight loss	2 (0.7)
	Rabies vaccine	96 (33.1)

Abbreviation: VBDs, vector‐borne diseases.

In the present study, ectoparasites were collected from an overall of 407 animals (297 dogs and 110 cats) from 21 Peasant Association (PA) (the lowest administrative unit) found in Arba Minch town (*n* = 8), Arba Minch Zuria district (*n* = 6), Chencha town (*n* = 4) and Gerese district (*n* = 3). The details of the sampling effort are reported in Table 2.

**TABLE 2:** Number of dogs and cats sampled according to district, sex, age class and provenance area.

	Cat				Dog				
District	Female		Male		Female		Male		Total
	Adult	Young	Adult	Young	Adult	Young	Adult	Young	
**Arba Minch town**									
Lowland (urban)	23	7	7	3	33	9	71	41	194 (47.7%)
**Arba Minch Zuria**									122 (30.0%)
Lowland (rural)	7	1	2	1	17	6	16	8	58 (14.0%)
Midland (rural)	6	2	9	4	12	5	20	6	64 (15.7%)
**Chencha town**									
Highland (urban)	4		2		4	1	4	3	18 (4.4%)
**Gerese**									73 (17.9%)
Highland (rural)	1		1		2	4	3	4	15 (3.7%)
Highland (urban)	14	6	7	3	9	2	8	9	58 (14.3%)
Sub‐total	55	16	28	11	77	27	122	71	
**Total**	**110**				**297**				**407**

### 3.1 Ectoparasites collected from dogs and cats

During the study period, a total of eight genera of ectoparasites were identified comprising five tick species (*Rhipicephalus sanguineus, Rhipicephalus pulchellus, Rhipicephalus praetextatus, Amblyomma variegatum* and *Haemaphysalis leachi*), four flea species (*Ctenocephalides felis, Ctenocephalides canis, Echidnophaga gallinacea* and *Pulex irritans*) and two lice species (*Heterodoxus spiniger* and *Trichodectes canis*).

Results of the study revealed that ectoparasites in dogs were more prevalent and abundant than ectoparasites in sympatric cats that were sampled during the same period. In particular, the prevalence for tick species in dogs was 36.7% compared to 2.7% in cats, with statistically significant difference. Similarly, the prevalence for fleas was much higher (*p* < 0.001) in dogs (69.7%) as compared to cats (21.8%), although in dogs, the prevalence for lice was 4.7%, and lice was totally absent in cats (Table 3). Considering the prevalence of the different species, *Rh. sanguineus* and *A. variegatum* were the more prevalent among dogs, whereas only the tick *H. leachi* was found in few cats. *H. spiniger* was the most prevalent lice species in dogs. *E. gallinacea* was the only flea species most prevalent in cats as compared to dogs (Table 3).

**TABLE 3:** Comparison of prevalence values of different ectoparasites between dogs and cats.

	Dogs (*n* = 297)		Cats (*n* = 110)		*p*‐Value
Parasite species	Pos	Prev (%)	Pos	Prev (%)	
Ticks (overall)	109	36.7	3	2.7	<0.001
* Rhipicephalus sanguineus*	42	14.1	0	0.0	<0.001
* Rhipicephalus pulchellus*	10	3.4	0	0.0	>0.05
* Amblyomma variegatum*	68	22.9	0	0.0	<0.001
* Rhipicephalus praetextatus*	12	4.0	0	0.0	0.042
* Haemaphysalis leachi*	26	8.8	3	2.7	0.048
Fleas (overall)	207	69.7	24	21.8	<0.001
* Pulex irritans*	1	0.3	0	0.0	>0.05
* Ctenocephalides felis*	206	69.4	17	15.5	<0.001
* Ctenocephalides canis*	3	1.0	0	0.0	>0.05
* Echidnophaga gallinacea*	4	1.3	8	7.3	0.004
Lice (overall)	14	4.7	0	0.0	0.014
* Heterodoxus spiniger*	12	4.0	0	0.0	0.042
* Trichodectes canis*	2	0.7	0	0.0	>0.05

The mean abundance values (Table 4) were generally low (i.e. less than one ectoparasite/host) for all species, apart from the flea *C. felis* in dogs, the mean number of which was 7.0 fleas/host. In most cases, only adult ticks were found, apart from *Rh. sanguineus*, the larval stages (nymphs and larvae) of which were collected with similar frequency than adults, and from *A. variegatum*, which was infesting dogs mainly during the nymphal stage. The higher presence of the flea *E. gallinacea* in cats was confirmed also by the significantly higher (*p* = 0.002) abundance in this host species, compared to dogs, on which the hen flea was only sporadically encountered (Table 4).

**TABLE 4:** Number of collected specimens of the different species of ectoparasites (according to the development stage) and significant difference in the mean abundance between dogs and cats.

			Dogs (*n* = 297)		Cats (*n* = 110)		*p*‐Value (Mann–Whitney *U*‐test)
Parasite species			*N* collected specimens	Mean abundance	*N* collected specimens	Mean abundance	
Ticks	*Rhipicephalus sanguineus*	**Total**	**216**	**0,7**	**0**		
		Male	70		0		
		Female	57		0		
		Nymph	76		0		
		Larvae	13		0		
	*Rhipicephalus pulchellus*	**Total**	**16**	**0,1**	**0**		
		Male	9		0		
		Female	7		0		
	*Amblyomma variegatum*	**Total**	**146**	**0,5**	**0**		
		Male	4		0		
		Female	0		0		
		Nymph	136		0		
		Larvae	6		0		
	*Rhipicephalus praetextatus*	**Total**	**17**	**0,1**	**0**		
		Male	9		0		
		Female	8		0		
	*Haemaphysalis leachi*	**Total**	**60**	**0,2**	**3**	**0,0**	
		Male	17		0		
		Female	43		3		
Fleas	*Pulex irritans*	**Total**	**1**	**0,0**	**0**		
		Males	1		0		
	*Ctenocephalides felis*	**Total**	**2076**	**7,0**	**29**	**0,3**	***P* < 0.001**
		Male	541		7		
		Female	1535		22		
	*Ctenocephalides canis*	**Total**	**29**	**0,1**	**0**		
		Male	22		0		
		Female	7		0		
	*Echidnophaga gallinacea*	**Total**	**31**	**0,1**	**78**	**0,7**	***p* = 0.002**
		Males	6		11		
		Females	25		67		
Lice	*Heterodoxus spiniger*	**Total**	**41**	**0,1**	**0**		
		Males	24		0		
		Females	17		0		
	*Trichodectes canis*	**Total**	**7**	**0,0**	**0**		
		Males	3		0		
		Females	4		0		

### 3.2 Risk factors for ectoparasite presence

Differences among subgroups of dogs identified by risk factors and statistical significance are reported in Table 5.

**TABLE 5:** Prevalence (P) of the main ectoparasites for the subgroups of dogs (*n* = 297) and statistical differences (*p*‐value in bold when significant).

			Ticks												Fleas						Lice		
			Tick (overall)			*Rhipicephalus sanguineus*			*Amblyomma variegatum*			*Haemaphysalis leachi*			Flea (overall)			Lice (overall)					
Factor	Variable	Tested	Pos	P (%)	*p*‐Value	Pos	P (%)	*p*‐Value	Pos	P (%)	*p*‐Value	Pos	P (%)	*p*‐Value	Pos	P (%)	*p*‐Value	Pos	P (%)	*p*‐Value	Pos	P (%)	*p*‐Value
Urbanization	Urban	194	62	32.0	**0.020**	42	21.6	**<0.001**	23	11.9	**<0.001**	14	7.2	>0.05	127	65.5	**0.029**	126	64.9	**0.024**	10	5.2	>0.05
	Rural	103	47	45.6		0	0		45	43.7		12	11.7		80	77.7		80	77.7		4	3.9	
Agroecology	Lowland	201	79	39.3	**<0.001**	42	20.9	**<0.001**	41	20.4	**<0.001**	13	6.5	**<0.001**	134	66.7	>0.05	133	66.2	>0.05	9	4.5	>0.05
	Midland	43	27	62.8		0	0		25	58.1		12	27.9		31	72.1		31	72.1		2	4.7	
	Highland	53	3	5.7		0	0		2	3.8		1	1.9		42	79.2		42	79.2		3	5.7	
Age class	Young	150	48	32.0	>0.05	16	10.7	>0.05	32	21.3	>0.05	5	3.3	**0.001**	104	69.3	>0.05	103	68.7	>0.05	7	32.0	>0.05
	Adult	147	61	41.5		26	17.7		36	24.5		21	14.3		103	70.1		103	70.1		7	41.5	
Sex	Female	104	39	37.5	>0.05	14	13.5	>0.05	25	24.0	>0.05	10	9.6	>0.05	80	76.9	**0.047**	80	76.9	**0.038**	3	37.5	>0.05
	Male	193	70	36.3		28	14.5		43	22.3		16	8.3		127	65.8		126	65.3		11	36.3	
Life style	Indoor	15	11	73.3	**0.003**	6	40.0	**0.003**	5	33.3	>0.05	3	20.0	>0.05	9	60.0	>0.05	9	60.0	>0.05	1	6.7	>0.05
	Outdoor	282	98	34.8		36	12.8		63	22.3		23	8.2		198	70.2		197	69.9		13	4.6	

Results of the present study indicate that ticks on dogs showed an opposite trend related to urbanization; *A. variegatum* was most abundant in dogs from rural areas (43.7% vs. 11.9%), whereas on the contrary, *Rh. sanguineus* and *Rh. pulchellus* were present only in urban areas. The prevalence of fleas appears to be higher in rural areas as compared to urban areas (*p* = 0.029), and this is mainly due to *C. felis*, the predominant species of fleas. Based on agroecology, a significantly lower prevalence of ticks was encountered on dogs living in highland (5.7%) agroecology than those dogs living in other agroecologies. Both *A. variegatum* and *H. leachi* were with significantly higher prevalence than in the midland, whereas *Rh. sanguineus* was found only in the lowland agroecology. A statistically significant difference was not found in the prevalence of both fleas and lice among agroecological areas. Statistically significant difference was not observed between adult and young dogs in the prevalence of different types of ectoparasites, a part from *H. leachi* that was predominantly found in older dogs. Likewise, significant difference was not recorded between male and female dogs in the prevalence of different ectoparasites, with the only exception of the flea *C. felis* that seems to prefer female hosts (*p* = 0.038). Finally, *Rh. sanguineus* was more prevalent in dogs which lived in indoor environment (40.0%).

## 4 DISCUSSION

Several surveys have been conducted on ectoparasites of food‐producing animals in African countries, including Ethiopia. However, comparatively little information is available on the occurrence and prevalence of ectoparasites of dogs and cats; for instance, very few previous studies have documented ectoparasite infestation in sympatric dog and cat populations in central parts of Ethiopia (Kumsa & Mekonnen, 2011; Kumsa et al., 2019). Therefore, the present study investigated the comparative epidemiological roles played by dogs and cats living in sympatry in harbouring different species of ectoparasites in four districts of Gamo Zone in Southern Ethiopia and provided useful information.

Results of the present study showed the collected ectoparasites were identified in eight genera, comprising five species of ticks (*Rh. sanguineus, Rh. pulchellus, Rh. praetextatus, A. variegatum* and *H. leachi*), four flea species (*C. felis, C. canis, E. gallinacea* and *P. irritans*) and two lice species (*H. spiniger* and *T. canis*). This study supported several pieces of evidence which attest that a higher prevalence and abundance of ectoparasite species were observed on dogs as compared to sympatric cats sharing the same environment (Kumsa & Mekonnen, 2011; Kumsa et al., 2019; Morelli et al., 2021). In particular, the prevalence of tick species was significantly higher in dogs (36.7%) as compared to cats (2.7%), and similarly, the prevalence of fleas was much higher in dogs (69.7%) than in cats (21.8%). On the other hand, cats were mostly affected by fleas, whereas ticks seem to infest this host species only sporadically. The study indicated a low prevalence (4.7%) of lice on dogs, which was totally absent in cats.

The overall prevalence of ticks on the dogs of the present study is in line with what was reported previously in other African countries (Nigeria by Ekanem et al., 2010), and Asian countries (India by Abd Rani et al., 2011 and Pakistan by Jafri & Rabbani, 1999) were most probably attributed to similar management conditions. For similar reasons, a higher prevalence of ticks on dogs, ranging from 45% to 55% and even exceeding 90%, was recorded in some areas of Nigeria (Agbolade et al., 2008). On the contrary, as compared to the present study, a lower overall prevalence of even less than 20% of ticks on dogs was reported in European countries (Cassini et al., 2009; Smith et al., 2011). This difference is probably due to the wide use of preventive measures (i.e. collars and spot‐on devices) in owned dogs.

Findings of the high overall prevalence of *A. variegatum* and *Rh. sanguineus* than the other tick species on dogs of the study area are in line with what was already reported in previous study in Nigeria (Elelu et al., 2022). However, higher counts of nymphal and larval stages (142/146, 97.3%) than the adult counts were recorded in *A. variegatum* tick specimens, whereas on the contrary, adults were predominant (127/216, 58.7%) in *Rh. sanguineus* ticks collected from dogs of the present study. Besides, *A. variegatum* was the most abundant tick in dogs from rural areas (43.7%), whereas *Rh. sanguineus* and *Rh. pulchellus* were present only in urban areas, which is in agreement with the observation of higher prevalence of *Rh. sanguineus* in urban areas as compared to the rural ones (Neves et al., 2004; Shimada et al., 2003). As suggested by Heylen et al. (2021), this could be due to the behaviour of *Rh. sanguineus* ticks, which prefer man‐made constructions to hide in cracks and crevices to oviposit or moult and consequently infest dogs near these structures in urban areas. Dogs act as a preferred host for nymphal and larval stages of *A. variegatum*, mostly in rural areas where other animal species that usually harbour the adult stages of this tick species (e.g. domestic and wild herbivores) are more abundant and live in sympatry with dogs, which is the most probable explanation for the above‐mentioned observation of the present study. On the other hand, the two tick species known to be strongly associated with the dog, that is the brown dog tick (*Rh. sanguineus*) and the African dog tick (*H. leachi*), were indeed commonly found on dogs, although not with high abundance (on average, less than 1 specimen per host). The other two species of the genus *Rhipicephalus* were sporadically found only in dogs, suggesting a marginal epidemiological role of both dogs and cats in the life cycle of these tick species. *H. leachi* was the only tick species collected from cats of the present study, which was most probably due to a mix of natural resistance and specific behaviour of cats as hosts of these ticks.

Observation of fleas as the most prevalent and abundant ectoparasite group on both dogs and cats in the present study, especially with the prevalence of 50% *C. felis* was in line with other previous studies conducted on dogs and cats in Jimma town (Tadesse et al., 2019), in Gondor town (Tesfaye & Chanie, 2011), in Hawassa town (Kumsa & Mekonnen, 2011) and in Bishoftu (Kumsa et al., 2019) in different parts of Ethiopia, all of which reported *C. felis* as the most prevalent ectoparasite, followed by *C. canis*. This confirmed that in Ethiopia, *C. felis* is the predominant flea species found on both dogs and cats, replacing *C. canis* on domestic dogs, which is generally regarded as the predominant flea species in many countries around the world. This might reflect the wider range of environment suitable for the survival, development and reproduction of C*. felis* as compared to all the other flea species.

The lower prevalence and burden of *C. felis* on cats than on dogs recorded in the present study is in agreement with most of the previous studies, although values may vary greatly among different studies according to geographical areas and inclusion criteria. For instance, a study conducted in free‐roaming domestic cats in the central USA reported 72% cats with flea infestation. Likewise, a previous study on shelter cats evidenced flea infestation in 73% of cats (Thomas et al., 2016), which is a much higher prevalence than the prevalence of the present study. Similarly, a higher prevalence of *C. felis* on cats than in the present study was reported from Ethiopia (Kumsa et al., 2019). On the other hand, similar to the present study, a prevalence of 15% fleas on cats was reported by Beugnet et al. (2014). The higher prevalence and abundance of *E. gallinacea* on cats than on dogs in the present study may reflect the greater association and exposure of cats to chickens or wild birds than dogs, as has been suggested earlier by others (Kumsa et al., 2019). It has been accepted that *E. gallinacea* is a species frequently found on birds, and occasionally reported on dogs and cats, due to transient infestations through contact with infested birds. Significantly, the higher prevalence and abundance of *P. irritans* on dogs than on cats may reflect the closer association of dogs to humans than in cats, as has been suggested before (Kumsa et al., 2019).

The observation of a low prevalence of lice species, namely *H. spiniger* (4.0%) and *T. canis* (0.7%), only on dogs is consistent with the already‐known limited diffusion of lice in general. On the contrary, Opeyemi et al. (2019) reported a high prevalence of *H. spiniger* in Nigeria. This might be attributed to several factors, including differences in the time of the study and animal management among these study areas. However, other studies conducted in different countries and socio‐economic settings found different results. In particular, the low prevalence of lice infestation was observed in urban cats in Brazil (Mendes‐de‐Almeida et al., 2011) and in a nationwide survey in Italy (Genchi et al., 2021).

The observation of a higher prevalence of fleas and some tick species on dogs living in rural areas as compared to those living in urban areas (*p* = 0.029) is in line with the previous observation in Sub‐Saharan Africa, where higher prevalence of ectoparasites in dogs was found in rural areas of Nigeria, Ghana, Tanzania and Kenya as compared to urban areas (Heylen et al., 2021). In general, rural areas may be considered a major risk for interspecific transmission of flea and tick species due to the presence of a higher number of domestic animals. This aspect can increase the chance of infestation of dogs due to the presence of ectoparasite species involving also other hosts in their life cycle, such as the case of *A. variegatum* in this study.

Significantly  lower prevalence of ticks was recorded on dogs which live in highland agroecology, suggesting that colder climate delay the completion of the life cycle of ectoparasites, in which the developmental stages of ticks stay for prolonged time. In support of the observation of the present study, it is also a well‐known fact that a higher prevalence and greater biodiversity of ticks were recorded on dogs from lowland and midland agroecologies than those dogs from highlands (Bermúdez & Miranda 2011). However, on the other hand, statistically significant differences were not observed in the prevalence of fleas and lice of dogs among different agroecological zones of the study area.

In this study, the prevalence of *H. leachi* was significantly higher in adult than young dogs, which is in agreement with the previous study in Iran by Mosallanejad et al. (2012) that reported a higher prevalence of ectoparasites in adult dogs as compared to the young ones. On the contrary, higher incidence among dogs younger than 1‐year old was reported in India (Raut et al., 2006). The prevalence of *C. felis* was significantly higher in female (*p* = 0.038) than male dogs, which is in line with previous reports in different countries of the world (Arong et al., 2011; Dantas‐Torres et al., 2009; James‐Rugu & Jidayi, 2004; Ul‐Hasan et al., 2012). On the other hand, unlike the present study, the same prevalence of *C. felis* in male and female dogs was reported in Nigeria (Agu et al., 2020). In contrast, other studies reported a higher prevalence of *C. felis* in male than female dogs (Jamshidi et al., 2012; Mosallanejad et al., 2012).

In conclusion, this study investigated the presence of ectoparasites in dogs and cats living in sympatry in the urban and rural areas of Gamo Zone, Ethiopia. The dogs and cats in this study share a common environment with humans, which makes them the key reservoirs for ectoparasites. This role has the potential to infest humans living in the same surroundings and transmit zoonotic VBDs. The data presented in this study are important for building knowledge about the occurrence of ectoparasites infesting dogs and cats in Southern Ethiopia and, consequently, designing future surveillance and prevention strategies. More importantly, the present study disclosed some epidemiological aspects such as the different distributions among dogs and cats and between different environmental settings of tick species. According to our questionnaire survey, most of the pet owners (*n* = 256/290, 88.3%) in the area had no knowledge about ectoparasites and VBDs of dogs and cats, and the majority of them (*n* = 188/290, 64.8%) never visited a veterinary clinic. This general absence of knowledge on the importance of dog and cat health status in a public health perspective, in combination with the finding of medium/high burden of ectoparasites acting as vector for important zoonotic VBDs (i.e. *Rh. sanguineus* and *C. felis*) suggests the need to carry out awareness creation campaign in the area and to strengthen veterinary care and services of dogs and cats of the present study area.

## AUTHOR CONTRIBUTIONS

*Conceptualization; data curation; formal analysis*: Hana Taddesse, Bersissa Kumsa and Rudi Cassini. *Investigation; methodology*: Hana Taddesse and Bersissa Kumsa. *Project administration*: Bersissa Kumsa and Rudi Cassini. *Resources*: Bersissa Kumsa. *Software*: Rudi Cassini. *Supervision; writing—review and editing*: Bersissa Kumsa, Marika Grillini, Antonio Frangipane di Regalbono and Rudi Cassini. *Validation*: Bersissa Kumsa, Marika Grillini, Antonio Frangipane di Regalbono and D.A. (Dinka Ayana) *Writing—original draft*: Hana Taddesse. All authors have read and agreed to the published version of the manuscript.

## CONFLICT OF INTEREST STATEMENET

The authors declare no conflicts of interest.

## FUNDING INFORMATION

None.

## ETHICS STATEMENT

Ethical approval for the collection of lice, fleas and ticks from dogs and cats from the study area was obtained from the Animal Research Ethics Board (Agreement No. VM/ERC/01/13/021) of the College of Veterinary Medicine and Agriculture of Addis Ababa University. All necessary permits were obtained, including permission of administration and agricultural office of the districts and verbal consent from each animal owner.

### PEER REVIEW

The peer review history for this article is available at https://publons.com/publon/10.1002/vms3.1413.

## Data Availability

The files containing the data supporting the reported results can be requested directly from the corresponding author.
